# Consensus Review and Definition of Nonallergic Rhinitis With a Focus on Vasomotor Rhinitis, Proposed to Be Known henceforth as Nonallergic Rhinopathy: Introduction to Part 2

**DOI:** 10.1186/1939-4551-2-8-155

**Published:** 2009-08-15

**Authors:** Michael A Kaliner, Judith R Farrar

**Affiliations:** 1 Institute for Asthma and Allergy, Chevy Chase, MD, Washington, DC; 2 LifeSciences Press, Washington, DC

**Keywords:** nonallergic vasosmotor rhinitis, nonallergic rhinitis, vasomotor rhinitis, idiopathic rhinitis, nonallergic rhinopathy

## Abstract

"Nonallergic vasomotor rhinitis" (also referred to as nonallergic rhinitis and/or idiopathic rhinitis) is a term that has been used to describe a common nasal condition of unclear pathophysiology. Clinical options for patients are limited by a lack of straight-forward diagnostic criteria and poorly defined and heterogeneous populations in clinical trials. A roundtable conference convened in December 2008 addressed these challenges. Part 1 of the proceedings of that meeting provided a consensus definition of "nonallergic rhinopathy," proposed to replace the former terms above based on the clinical characteristics of the disease, which were described in individual papers. Part 2 of the proceedings uses the revised definition for a consensus discussion on appropriate criteria for enrolling subjects in future clinical studies of the efficacy of potential treatments for this disease.

## Introduction

"Nonallergic vasomotor rhinitis" (also referred to as non-allergic rhinitis and/or idiopathic rhinitis) is a term that has been used to describe a common nasal condition of unclear pathophysiology. Clinical options for patients are limited by a lack of straightforward diagnostic criteria and poorly defined and heterogeneous populations in clinical trials. A roundtable conference convened in December 2008 addressed these challenges. Chaired by Michael A. Kaliner, MD, the consensus meeting included James N. Baraniuk, MD; Michael Benninger, MD; Jonathan A. Bernstein, MD; Phil Lieberman, MD; Eli O. Meltzer, MD; Robert M. Naclerio, MD; and Russell A. Settipane, MD. Each expert participant presented a State of the Art paper on a topic related to what is currently known about the pathophysiology, epidemiology, diagnostic criteria, and treatment options. The presentations were followed by discussion and counterpoint leading to 2 consensus statements--one on the definition and the other on the study criteria. Our goal was to work toward improving understanding of the phenotypes to identify acceptably homogeneous populations for studying the underlying mechanisms and developing better therapies. The proceedings of that meeting are summarized herein and include the findings, discussion, and recommendations.

The proceedings have been divided into 2 parts. Part 1, published in the June issue of the journal started with an overview of the classification of nonallergic rhinitis syndromes and the proposal to change the terminology to reference these as "nonallergic rhinopathy;" it concluded with the after consensus definition of the new term. "Nonallergic rhinopathy (NAR) is a chronic nasal condition with symptoms that may be perennial, persistent, intermittent or seasonal and/or elicited by recognized triggers. There are a well recognized set of clinical exposures that lead to the symptoms, predominantly congestion, rhinorrhea and postnasal drip." NAR is defined by clinical characteristics that are described in detail in Part 1 of these proceedings.

The variable symptomatology and triggers of NAR have led to confusion about specific diagnostic testing and appropriate treatment options. These challenges are discussed in Part 2 of the proceedings, presented here, and include review of the major classes of medications used to treat these patients. Discussion culminated in a consensus statement and specific suggestions for acceptable diagnostic criteria to permit precise identification of patients for participation in future clinical trials of potential therapies for this disease. The criteria are based on the clinical characteristics described in the first part of the proceedings.

As the proceedings from the meeting are read, please keep in mind that the older terminology (vasomotor rhinitis, nonallergic rhinitis) remains in the individual papers as they were written and submitted before the consensus discussion.

## End Note

Presented at a roundtable conference held in December 2008 in Washington, DC. The meeting was sponsored by the TREAT Foundation (Washington, DC) and supported through an unrestricted educational grant from Meda Pharmaceuticals. The funding company did not have any input into the development of the meeting or the series, and the company was not represented at the roundtable meeting. 

